# Synchronous mixed medullary‐papillary carcinoma and papillary multifocal carcinoma of the thyroid with cervical lymph node metastases

**DOI:** 10.1002/ccr3.7433

**Published:** 2023-05-29

**Authors:** Raquel Lalanda, David Aparício, João Boavida, Dolores Lopes Presa, Lucas Batista, Luís Miranda

**Affiliations:** ^1^ General Surgery Department – Endocrine Surgery – Centro Hospitalar Universitário Lisboa Norte EPE Hospital de Santa Maria Lisbon Portugal; ^2^ Pathology Department – Centro Hospitalar Universitário Lisboa Norte EPE Hospital de Santa Maria Lisbon Portugal

**Keywords:** central neck lymph node dissection, mixed medullary‐papillary carcinoma, multifocal papillary thyroid carcinoma

## Abstract

**Key Clinical Message:**

The occurrence of simultaneous multifocal papillary thyroid carcinoma and mixed medullary‐papillary carcinoma, as far as we know, has not been previously described. We suggest the surgical approach to be driven by the medullary component.

**Abstract:**

Patient underwent total thyroidectomy with central compartment lymph node dissection. Histological examination revealed a simultaneous multifocal papillary thyroid carcinoma and mixed medullary‐papillary carcinoma. He was disease‐free at 1‐year‐follow‐up.

## Graphical abstract

Histological examination revealed a simultaneous mixed medullary‐papillary carcinoma (in the picture) and a multifocal papillary thyroid carcinoma.

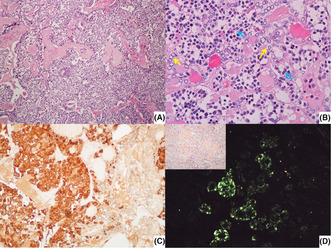

## 1 CASE PRESENTAION

A 64‐year‐old female with medical history of hypertension, diabetes and non‐toxic multinodular goiter had increased calcitonin levels (1499 pg/mL) with normal thyroglobulin levels (1.49 ng/mL) and slightly elevated TSH levels (4.32uU/mL). Ultrasound revealed two isoechoic nodules measuring 16 and 19 mm (TIRADS 3) with no highly suspicious features and no suspicious cervical lymph nodes. Fine‐needle aspiration of thyroid nodules was suspected of medullary carcinoma. There was no childhood history of head and neck radiation nor family history of thyroid cancer or any MEN syndrome.

**FIGURE 1:**
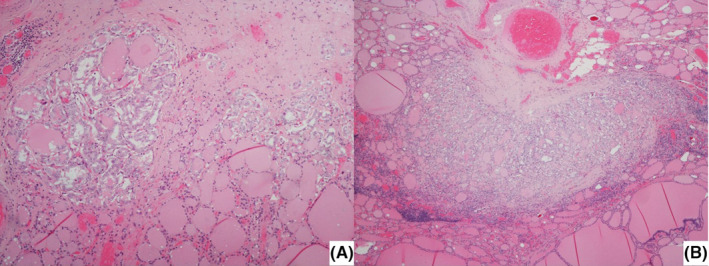
(A, B) Low power view of two foci of papillary microcarcinoma on the left and right lobes, respectively. (H & E, 40×);

**FIGURE 2:**
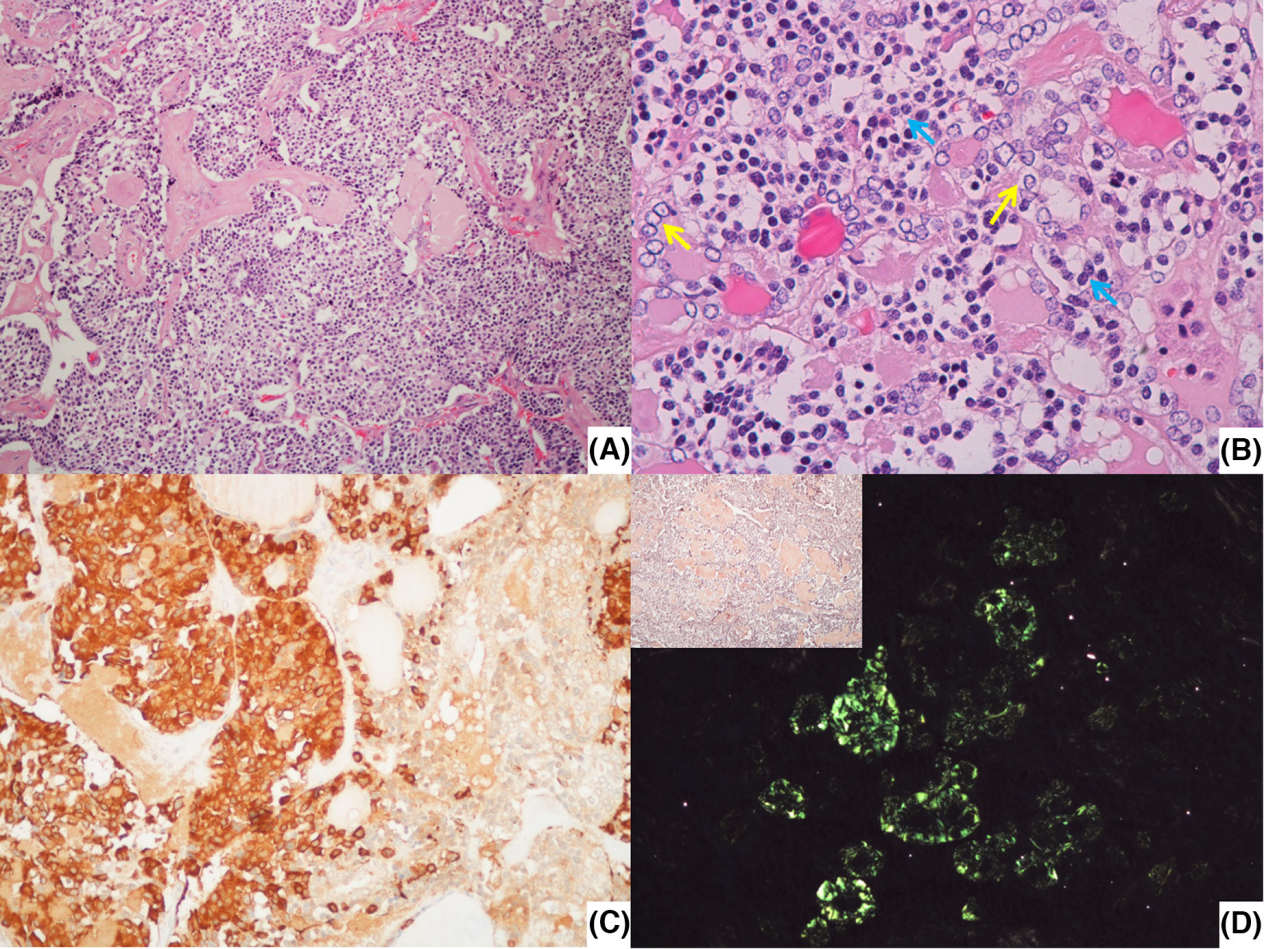
(A) Low power view of mixed medullary‐papillary carcinoma on the right lobe (H & E, 100×); (B) Detail of the nuclei of the cells composing the tumor in A (small and round cells with clumped chromatin and finely granular cytoplasm—blue arrows—and cells with papillary features, that is, empty appearance of the nucleoplasm and nuclear irregularity and overlapping – yellow arrows) (H & E, 400×); C – Immunohistochemical staining for calcitonin highlights the medullary component (200×); D – Amyloid deposition, confirmed in polarized light, was already apparent using Congo red histochemistry (inset) (200×).

Patient underwent total thyroidectomy with central compartment lymph node dissection. Surgery was uneventful and patient was discharged on first postoperative day. Histological examination revealed a bilateral papillary multifocal carcinoma of the thyroid, pT1a(m) (Figure 1) and a mixed medullary‐papillary carcinoma of the thyroid, with 1,7 cm, pT1b, on the right lobe (Figure 2). Two out of twenty‐six isolated central lymph nodes had metastases from the papillary carcinoma (compartment VI), and three had metastases from the medullary carcinoma (N1a). Parathyroid gland tissue, was found adjacent to both lobes on histological examination, due to surgical procedure it was identified as inferior parathyroid.

Patient had postoperative hypocalcemia (PTH levels of 3 pg/mL) that was successfully corrected with oral calcium and vitamin D supplementation. PTH levels remained low (<7 pg/mL) and she continued taking oral calcium supplementation and levothyroxine.

After surgery calcitonin levels dropped to 1,06 pg/mL and thyroglobulin levels dropped to 0,26 ng/mL. Patient was disease‐free at 1‐year‐follow‐up and remains under surveillance.

## 2 QUIZ QUESTION: IS THIS A FREQUENT OCCURRENCE?

Medullary thyroid carcinomas account for 5%–10% of all thyroid carcinomas.^1^ About 3%–5% of these cases may display mixed features, with either a follicular or papillary component in the primary tumor, in the metastases or in both.^1^ The existence of a mixed tumor is remarkable because the two cells derived are from different origin—neuroectodermal from the fourth brachial arch and foregut endodermal arising from the base of the tongue.^1^ In most cases, lymph node metastases were reported at diagnosis, as a single tumor cell population, or as a mixture of both components within the same lymph node.^2^

Papillary carcinoma accounts for more than 80% of all thyroid malignancies.^3^ The great majority of these tumors have good prognosis, unfortunately up to 50% relapse.^3^ Our patient had a papillary multifocal carcinoma with lymph node metastases which increases the risk of recurrence and requires watchful surveillance.^3^

The occurrence of the simultaneous papillary and mixed medullary‐papillary carcinoma, as far as we know, has not been previously described. We suggest that surgical approach should be driven by the medullary component. Total thyroidectomy plus central neck dissection plus lateral neck dissection in case of involved lymph nodes on radiological preoperative evaluation is accepted as being the first‐choice option.

## AUTHOR CONTRIBUTIONS

**Raquel Lalanda:** Conceptualization; data curation; formal analysis; investigation; methodology; resources; software; supervision; validation; visualization; writing – original draft; writing – review and editing. **David Aparício:** Conceptualization; formal analysis; investigation; methodology; supervision; validation; visualization; writing – review and editing. **João Boavida:** Investigation; resources; validation; visualization; writing – review and editing. **Dolores Lopez Presa:** Investigation; resources; supervision; validation. **Lucas Batista:** Resources; supervision; validation; visualization. **Luís Miranda:** Supervision; validation; visualization.

## FUNDING INFORMATION

None to declare.

## CONFLICT OF INTEREST STATEMENT

All authors declare no conflict of interests.

## CONSENT

Written informed consent was obtained from the patient to publish this case report in accordance with the journal's patient consent policy.

## Data Availability

The data that support the findings of this study are available from the corresponding author upon reasonable request.
